# Vesiculobullous eruption in an immunocompromised patient

**DOI:** 10.1016/j.jdcr.2025.05.053

**Published:** 2025-07-28

**Authors:** Cynthia Truong, Shadi Khalil, Amy K. Bieber, George Jour, Ata S. Moshiri

**Affiliations:** Ronald O. Perelman Department of Dermatology, New York University, New York, New York

**Keywords:** HHV-8, immunosuppression, Kaposi sarcoma, lymphangioma, organ transplantation

## History

**Fig 1:**
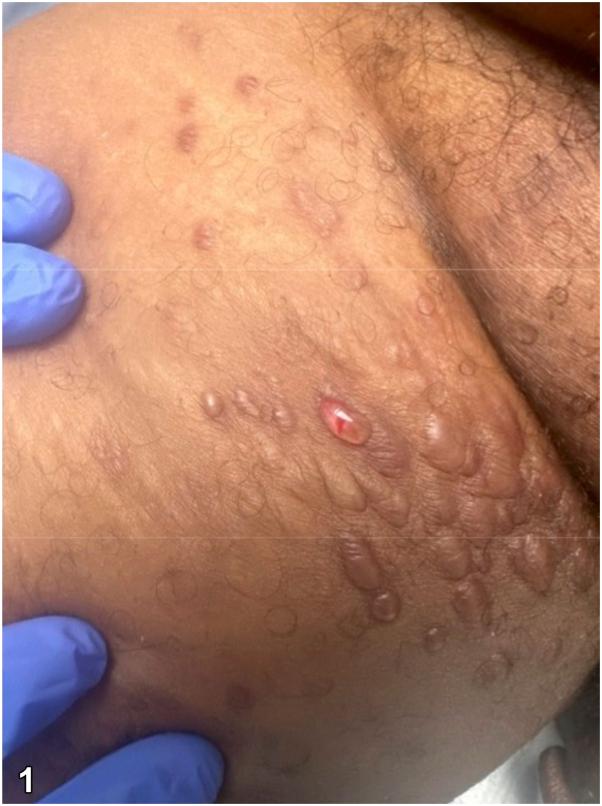

**Fig 2:**
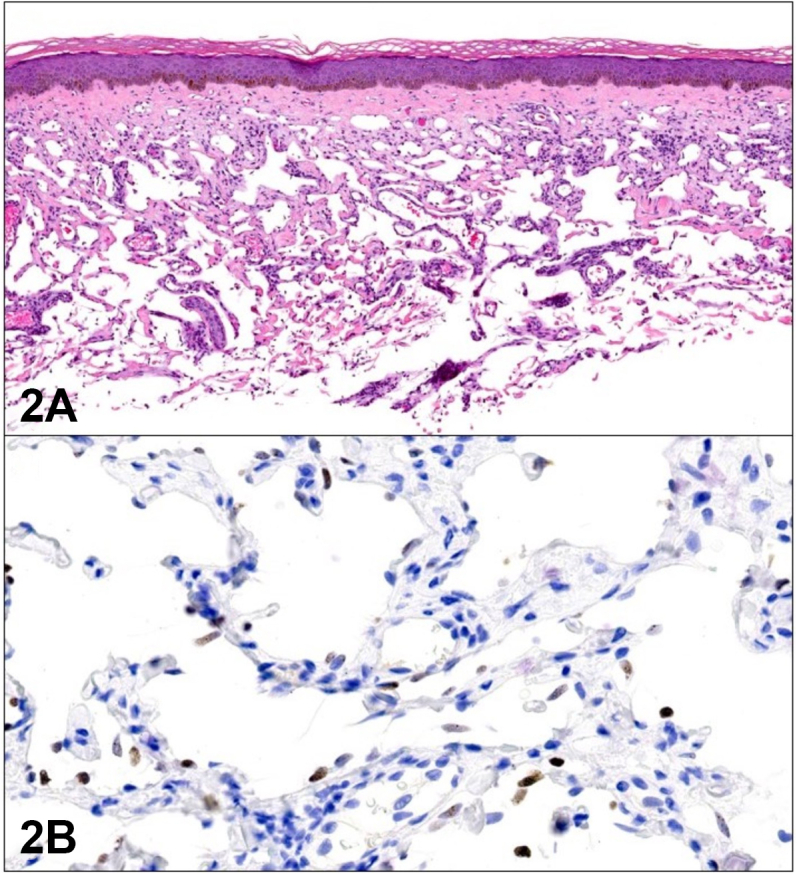

A 57-year-old male with cystic fibrosis status post lung transplant on tacrolimus presented with tense, clustered vesicles and bullae of the medial upper thighs (Fig 1). He was empirically treated with valacyclovir without improvement and developed progressive inguinal lymphadenopathy. Lymph node biopsy demonstrated uniform spindle cells, poorly formed slit-like ectatic vascular channels, and human herpesvirus-8 (HHV-8) immunoreactivity. Large lymphangioma-like vessels were noted peripherally. Skin biopsy obtained from the roof of a vesicular lesion demonstrated ectatic vascular channels without spindle cells (Fig 2, *A*) with positive expression of D2-40 and CD31. Nuclear immunoreactivity against HHV-8 was noted in a proportion of endothelial cell nuclei (Fig 2, *B*).

**Question 1: What is the most likely diagnosis?**

A. Kaposiform hemangioendothelioma (KHE)
B. Acquired lymphangiectasia
C. Targetoid hemosiderotic hemangioma (THH)
D. Lymphangioma-like Kaposi sarcoma (KS)
E. Lymphangioma circumscriptum (LC)

**Answers:**

A. Kaposiform hemangioendothelioma (KHE) – Incorrect. KHE is a vascular tumor of childhood. Histology can resemble KS, demonstrating coalescing spindled endothelial cells and slit-like vascular spaces; however, the vascular proliferations of KHE are often more lobular than KS and will not stain positive for HHV-8.
B. Acquired lymphangiectasia – Incorrect. Acquired lymphangiectasias are lymphatic malformations that present as secondary manifestations of various inflammatory, infectious, iatrogenic, or neoplastic processes. For example, metastatic breast carcinoma can present with intralymphatic metastasis and subsequent distortion of lymphatic channels. Lymphangioma-like KS, on the other hand, is a primary neoplasm caused by infection of endothelial cells with HHV-8.
C. Targetoid hemosiderotic hemangioma (THH) – Incorrect. THH often presents in young to middle-aged individuals and follows a benign course.^1^ Unlike KS, the endothelial cells of THH are more plump and epithelioid and often protrude into the lumen of the vessel.
D. Lymphangioma-like Kaposi sarcoma (KS) – Correct. KS is a vascular malignant tumor that is associated with HHV-8, a herpesvirus that infects a broad range of cell types including lymphocytes and endothelia. Lymphangioma-like KS is an uncommon presentation of KS. In our patient, the clinical presentation, including history of immunosuppression, can help suggest the correct diagnosis, although prior reported cases have shown predominantly papules, vesicles, and bullae.^2^
E. Lymphangioma circumscriptum (LC) – Incorrect. LC presents on the skin as grouped, grape-like, translucent vesicles that may appear red or violaceous if hemorrhage is present. The clinical and histopathologic presentation of LC may mimic that of lymphangioma-like KS; however, LC will not display HHV-8 immunoreactivity.

**Question 2: Which of the following strategies can be used to confirm the diagnosis?**

A. Biopsy of vesicle for direct immunofluorescence
B. Swab of base of deroofed vesicle for viral polymerase chain reaction (PCR)
C. Deep biopsy of vesicle for immunohistochemistry and tissue PCR
D. Serum HIV1/2 RNA and CD4+ T cell count
E. HHV-8 serology

**Answers:**

A. Biopsy of vesicle for direct immunofluorescence – Incorrect. While the clinical differential for this patient included primary blistering disorders, the pathophysiology of bullae in lymphangioma-like KS is marked lymphangiectasia resulting in bullous-appearing lesions. Thus, direct immunofluorescence would be negative.
B. Swab of base of deroofed vesicle for viral polymerase chain reaction (PCR) – Incorrect. Given the history of immunosuppression and the vesicular morphology of the rash, this patient was treated empirically with valacyclovir. Herpes simplex virus and varicella zoster virus DNA testing, however, was negative.
C. Deep biopsy of vesicle for immunohistochemistry and tissue PCR – Correct. In lymphangioma-like KS, HHV-8 immunoreactivity and spindle cells may be only focally present, and there is high probability of misclassification with inadequate sampling of the lesion. Early lesions tend to have few classic histopathologic features, while later lesions may be more diagnostic.^1^ To mitigate this, deep biopsies of vesicles should be performed for lesions where there is diagnostic concern. Furthermore, tissue PCR for HHV-8 may be needed in cases with negative HHV-8 immunohistochemistry, as multiple immunohistochemically negative cases have been reported.^3^^,^^4^
D. Serum HIV1/2 RNA and CD4+ T cell count – Incorrect. The 4 major subtypes of KS include classic, African endemic, transplant-associated, and acquired immune deficiency syndrome (AIDS)–associated. All patients with KS should be evaluated for infection with HIV; however, HIV positivity is not diagnostic of KS. In this case, the patient was iatrogenically immunosuppressed due to lung transplant. He was evaluated by hematology who recommended changing immunosuppression with potential future consideration of chemotherapy or radiation if lesions persisted.
E. HHV-8 serology – Incorrect. HHV-8 serology may be positive in patients without KS and is not a marker of active disease, therefore is not a useful confirmatory diagnostic test for lymphangioma-like KS.

**Question 3: Which of the following is NOT a described histologic variant of KS?**

A. Hypertrophic KS
B. Subepidermal bullous KS
C. Pyogenic granuloma-like KS
D. Ecchymotic KS
E. Verrucous KS

**Answers:**

A. Hypertrophic KS – Correct. Some rare variants of KS present as firm, hypertrophic, rubbery lesions. Histologically, these variants show dermal expansion of dense, hyalinized collagen and thus are classified into a subtype called keloidal, rather than hypertrophic, KS.
B. Subepidermal bullous KS – Incorrect. KS can present as tense vesicles and bullae, as in our patient. Subepidermal bullous KS is a variant in which accumulation of peritumoral edema in the superficial dermis leads to the development of tense bullous lesions.^2^ In contrast, in lymphangioma-like KS, patients clinically develop bullous-like lesions that represent the dilated and ectatic vascular channels seen histologically.^1^
C. Pyogenic granuloma-like KS – Incorrect. Pyogenic granuloma-like KS presents as an exophytic papule with an epidermal collarette that can often be confused for a true pyogenic granuloma. Lesions can become ulcerated and inflamed if traumatized.
D. Ecchymotic KS – Incorrect. Ecchymotic KS is a histologic variant seen in patients with AIDS-associated KS. Histologically, there is an intradermal proliferation of slit-like vascular endothelial spaces and marked red blood cell extravasation, which can obscure the histologic features of KS.
E. Verrucous KS – Incorrect. Verrucous, or hyperkeratotic, KS is a variant associated with chronic lymphedema in patients with AIDS-associated KS. Histologically, there is hyperkeratosis and verrucous epidermal acanthosis overlying the diagnostic slit-like vessels of KS seen in the dermis.

## Conflicts of interest

None disclosed.
